# Raising Dielectric Permittivity Mitigates Dopant‐Induced Disorder in Conjugated Polymers

**DOI:** 10.1002/advs.202101087

**Published:** 2021-08-11

**Authors:** Meenakshi Upadhyaya, Michael Lu‐Díaz, Subhayan Samanta, Muhammad Abdullah, Keith Dusoe, Kevin R. Kittilstved, Dhandapani Venkataraman, Zlatan Akšamija

**Affiliations:** ^1^ Electrical and Computer Engineering University of Massachusetts Amherst Amherst USA; ^2^ Chemistry University of Massachusetts Amherst Amherst USA; ^3^ Polymer Science and Engineering University of Massachusetts Amherst Amherst USA; ^4^ Institute for Applied Life Sciences University of Massachusetts Amherst Amherst USA

**Keywords:** conjugated polymer, dielectric, energetic disorder, P3HT, thermoelectric

## Abstract

Conjugated polymers need to be doped to increase charge carrier density and reach the electrical conductivity necessary for electronic and energy applications. While doping increases carrier density, Coulomb interactions between the dopant molecules and the localized carriers are poorly screened, causing broadening and a heavy tail in the electronic density‐of‐states (DOS). The authors examine the effects of dopant‐induced disorder on two complimentary charge transport properties of semiconducting polymers, the Seebeck coefficient and electrical conductivity, and demonstrate a way to mitigate them. Their simulations, based on a modified Gaussian disorder model with Miller‐Abrahams hopping rates, show that dopant‐induced broadening of the DOS negatively impacts the Seebeck coefficient versus electrical conductivity trade‐off curve. Increasing the dielectric permittivity of the polymer mitigates dopant‐carrier Coulomb interactions and improves charge transport, evidenced by simultaneous increases in conductivity and the Seebeck coefficient. They verified this increase experimentally in iodine‐doped P3HT and P3HT blended with barium titanate (BaTiO_3_) nanoparticles. The addition of 2% w/w BaTiO_3_ nanoparticles increased conductivity and Seebeck across a broad range of doping, resulting in a fourfold increase in power factor. Thus, these results show a promising path forward to reduce the dopant‐charge carrier Coulomb interactions and mitigate their adverse impact on charge transport.

## Graphical abstract

Organic electronic materials must be doped to increase their conductivity, but dopants increase energetic disorder and impede the motion of charge carriers. Here the authors show that the addition of nanoparticles having very high dielectric constants effectively screens the interaction between dopants and charge carriers, resulting in significant improvements in conductivity and the thermoelectric power factor.

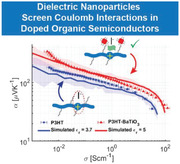

## 1 Introduction

Organic electronics have attracted intense research attention as they are environmentally friendly and solution‐processable, which makes them cost‐efficient. They are also lightweight and flexible, which allows for facile integration for wearables, medical monitoring, and internet‐of‐things electronics.^[^ ^1^ ^]^ Conjugated polymers are poor electronic conductors for two principal reasons: their morphology has a significant disorder and they do not have intrinsic free charge carriers. Therefore, conjugated polymers need to be doped, that is, oxidized or reduced, to increase the density of free carriers. These free carriers in the oxidized or reduced polymer impart electronic and optoelectronic properties of conjugated polymers that form the basis of organic electronics. The simple process of doping introduces complexities in the electronic structure because of the charge–charge Coulomb interactions between the dopant and the polymer. This long‐range interaction is much more pronounced in polymers than in inorganic semiconductors because of their low dielectric permittivity (<3).^[^ ^2^ ^]^ Inadequate screening of the dopant‐polymer interactions increases energetic disorder as it increases the width and alters the shape of the distribution of density of states (DOS).^[^ ^3^, ^4^, ^5^ ^]^ These factors suppress the density of free charge carriers by electrostatically binding the charge carriers to their conjugate dopant counterions, making doping less efficient. They also create deep traps, which adversely affect charge transport and thus the electronic and optoelectronic properties of the polymer.

The intrinsic DOS, which denotes the number of available states in a given energy range, typically follows a Gaussian distribution $g_{i}\left(E\right)\propto \exp\left(-E^{2}/2\Gamma_{E}^{2}\right)$, as prescribed in the Gaussian disorder model (GDM).^[^ ^6^ ^]^ The width of DOS (Γ_*E*_) is the “energetic disorder” arising from structural and morphological randomness.^[^ ^7^, ^8^ ^]^ When ionized dopants interact with carriers through long‐range Coulomb forces, it increases Γ_*E*_ and introduces a heavy tail in the DOS,^[^ ^4^ ^]^ which is particularly pronounced when dopants cluster together.^[^ ^5^ ^]^ A direct consequence of the changes in DOS is seen in the two principal measures of charge transport: conductivity (*σ*) and Seebeck coefficient (*α*), which is a measure of the open‐circuit voltage produced by a temperature gradient and is related to the average energy transported by each carrier. There is a dramatic flattening in the shape and a downward‐left shift of the entire *α*–*σ* curve.^[^ ^5^ ^]^ This directly impacts the electronic and optoelectronic properties of the polymer. For example, in thermoelectric materials a flattened *α*–*σ* curve limits the maximum power factor and thus the thermoelectric conversion efficiency.^[^ ^5^ ^]^ In organic photovoltaics, energetic disorder limits efficiency^[^ ^9^ ^]^ through open‐circuit voltage loss.^[^ ^10^ ^]^ For all these reasons, optimizing the properties of conjugated polymers for any application becomes empirically multivariate and complex.

Here we show, using a combined computational‐experimental study, that raising the dielectric constant of a polymer counteracts the dopant‐induced broadening of the DOS and results in a simultaneous increase in the Seebeck coefficient and electrical conductivity. Our simulations, which are based on the GDMbut modified to include electrostatic interactions between carriers and clustered dopants, use Pauli's master equation (PME) to calculate site occupational probabilities and simulate hopping of localized carriers from Miller‐Abrahams rates. We relate the dopant‐induced energetic disorder to a reduction in the Seebeck coefficient while deep traps in the heavy tail cause a collapse in conductivity. Increasing ϵ from 3 to 12 nearly restores the intrinsic DOS, resulting in a large increase in the power factor. Our experiments validate the computed results and show that we can increase the power factor by fourfold by incorporating 2% of BaTiO_3_ nanoparticles in poly(3‐hexylthiophene) (P3HT) films. Our method of incorporating additives with dielectric permittivity obviates the need for synthetic modifications and thus can be applied to wide range of polymers. Our results indicate that doped polymer composites with high dielectric permittivity are a fertile new avenue to decrease Coulomb interactions, improve charge transport in conjugated polymers, and develop high performance organic electronic materials.

## 2 Results and Discussion

### 2.1 Impact of Dopants on DOS and Transport

**Figure 1:**
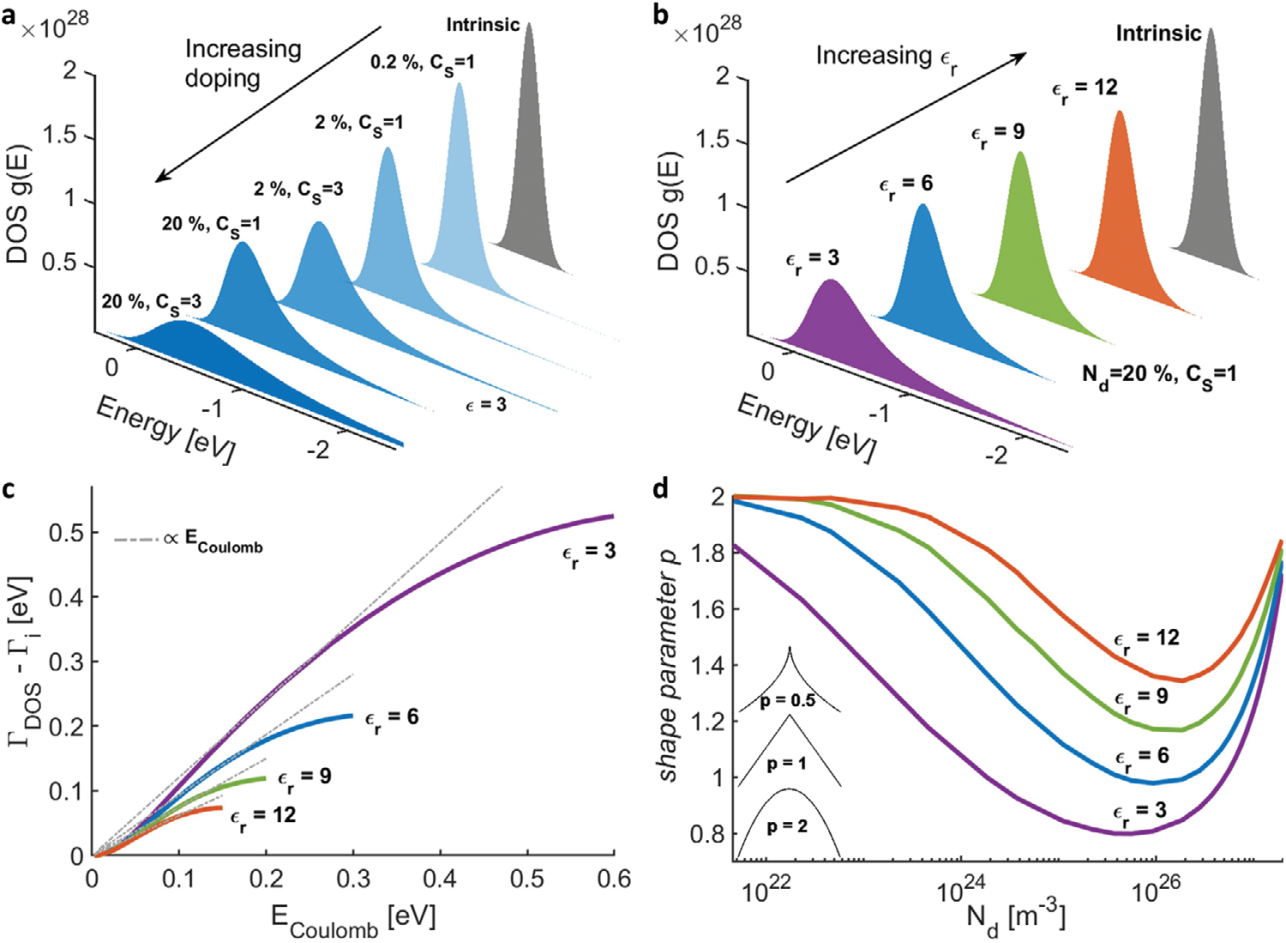
Increasing the dielectric permittivity counteracts the broadening of the DOS. a) The effect of doping and clustering on the DOS distribution with intrinsic Gaussian width of 100 meV. b) Increasing the dielectric constant counters the broadening of the DOS by mitigating the Coulomb carrier‐dopant interactions c) Additional energetic disorder caused by doping as a function of Coulomb energy for the standard (*ε*_*r*_ = 3) and elevated dielectric constants. The grey dashed lines are linear fits showing that the amount of energetic disorder closely follows the Coulomb interaction energy. d) Generalized Gaussian shape parameter *p* versus doping concentration showing that increasing the dielectric constant keeps the shape parameter more Gaussian (*p* ≈2) even at higher doping concentrations.

We calculated the DOS for doping concentration *N*_d_ in clusters having size *C*_s_, according to procedure in the Experimental Section and found that doping resulted in a heavy‐tailed distribution with a Gaussian core and a wide quasi‐exponential tail (**Figure** **1**a). Increasing dopant concentration lifted the long quasi‐exponential tail at the expense of the central Gaussian DOS; clustering of dopants further amplified this effect, as seen from the similarity between the DOS curves with cluster size *C*_s_ = 3 at 2% and *C*_s_ = 1 at 20% doping. Here *C*_s_ refers to the number of charges in each cluster while the percentage refers to the portion of simulated sites, on average spaced by 0.6 nm, that are occupied by carriers. Thermoelectric power factors are typically found to peak near 20% doping.^[^ ^11^ ^]^ Increasing the effective dielectric permittivity dramatically reduced the Coulomb interactions and minimized the tail (Figure 1b). A more general way to capture the impact of dopants is to extract the effective energetic disorder from each DOS curve. Energetic disorder is the standard deviation of the DOS g(E): $\Gamma_{E}^{2}=\int Eg\left(E\right)dE/\int g\left(E\right)dE$, which can be determined regardless of the shape of the DOS. The additional energetic disorder is caused by doping as it closely follows the Coulomb interaction energy with the nearest dopant (Figure 1c). Here *N*_d_ = *N*_s_ as *C*_s_ = 1 for all cases. Raising the dielectric permittivity lowered this additional dopant‐induced energetic disorder even at high doping concentrations, nearly restoring the intrinsic Gaussian DOS.

To capture the impact of doping and dielectric constant on the shape of the DOS, we liken it to a generalized Gaussian distribution (GGD), a parametric classification of symmetric distributions given by:^[^ ^12^ ^]^

(1) $$G\left(E\right)=\frac{p\;A\left(p,\Gamma_{E}\right)}{2\Gamma_{E}\gamma \left(1/p\right)}\exp\left\{-{\left[A\left(p\right)\left|E\right|/\Gamma_{E}\right]}^{p}\right\}$$

where $A\left(p\right)=\sqrt{\gamma (3/p)/\gamma (1/p)}$, *γ* denotes the gamma function, Γ_*E*_ is the standard deviation, and *p* is the shape parameter. A *p* value of 2 corresponds to a Gaussian distribution and smaller the *p* value is, the heavier the tail of the distribution, as illustrated in the inset of Figure 1d (Figure S1a, Supporting Information). For a given distribution, *p* can be estimated by finding the root of $M_{2p}/M_{p}^{2}-\left(1+p\right)=0$ using a secant method, where *M_r_* is the *r*th absolute moment of the GGD.^[^ ^12^ ^]^ We find that at low doping the shape parameter *p* is ≈2, indicating the DOS is more Gaussian and as we increase doping *p* decreases indicating that the tail gets heavier, with *p* reaching as low as 0.8 at very high doping values (Figure 1d). However, increasing the dielectric constant shifts the *p* values closer to 2 even at high doping concentrations, keeping the DOS more Gaussian.

**Figure 2:**
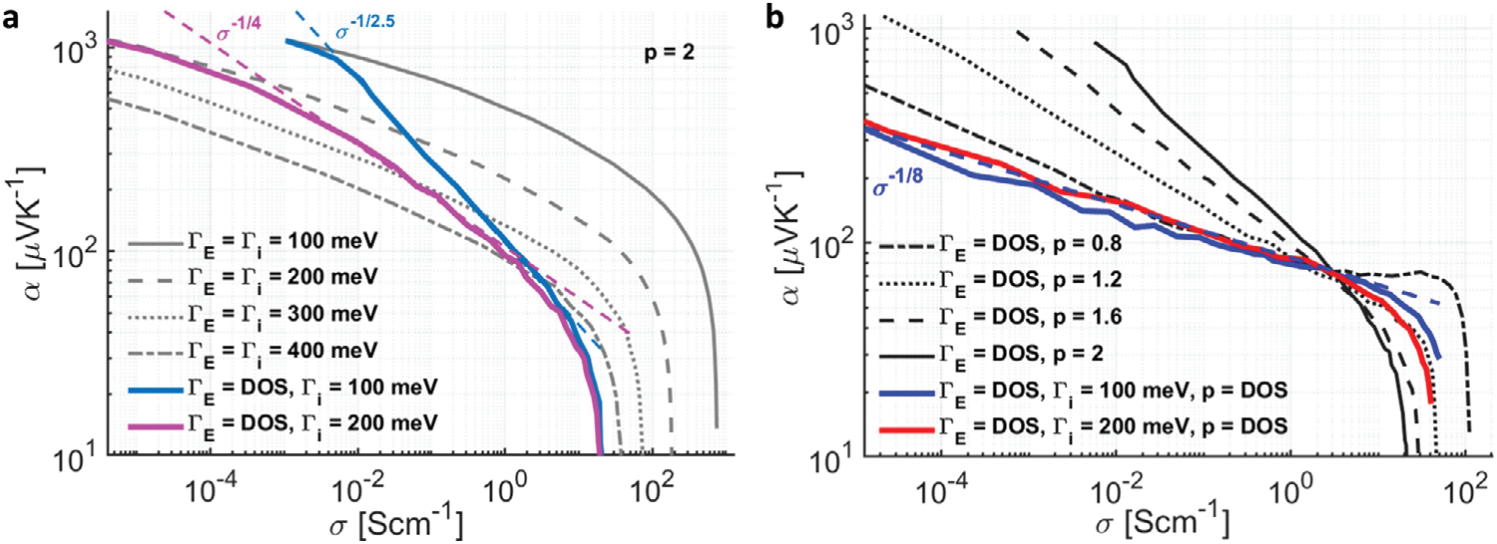
Effect of the width (Γ_*E*_) and shape parameter *p* of the DOS on the trend of the *α* versus *σ* curve. a) The *α* versus *σ* curves for GGD with fixed Γ_*E*_ (gray lines) and the *α* versus *σ* curve with Γ_*E*_ increasing with doping (pink and cyan lines). *p* is fixed to 2 for all the cases. b) The *α* versus *σ* curves for a DOS with shape parameter fixed to 0.8, 1.2, 1.6, and 2, and Γ_*E*_ increasing with doping (black lines). The blue and red lines show the standard case with DOS computed from Equation (5), where Γ_*E*_ and shape parameter *p* are both changing for Γ_*i*_ values of 100 and 200 meV, respectively.

Since doping affects both the width (Γ_*E*_) and shape (*p*) of the DOS distribution, we studied their individual impact on the *α* versus *σ* curve. First, we used a fixed Gaussian DOS (*p* = 2 in Equation (1)), keeping its width Γ_*E*_ constant across doping concentrations, and obtained the *α* and *σ* at various carrier densities by moving *E_F_* closer to the center. In this case, larger Γ_*E*_ shifted the *α* versus *σ* curve down (lower *α*) and left (lower *σ*), with minimal changes to its slope (grey lines in **Figure** **2**a and Figure S2a, Supporting Information). However, if we let the Γ_*E*_ increase with doping concentration by extracting it from the DOS (Equation (5)) but keep the shape Gaussian (*p* = 2), the resulting *α* versus *σ* exhibited a much higher slope (∝*σ* ^−1/2.5^), indicating that the doping‐induced Γ_*E*_, while detrimental to transport in general, had a net effect of lowering *α* at higher doping concentrations. This can be understood from the Mott formula^[^ ^13^, ^14^ ^]^ $\alpha =-\left(\frac{\pi^{2}}{3}\right)\left(\frac{k_{B}^{2}T}{q}\right)\frac{\partial }{\partial E}\ln\left[\sigma (T,E)\right]{|}_{E=E_{F}}$—using the Einstein relation for *σ*(*T*, *E*), we get $\alpha \propto \frac{d\ln[g(E)]}{dE}+g\left(E\right)\frac{d[\mu (E)]}{dn}$. When *µ*(*E*) is only weakly varying, the second term is small, resulting in *α*∝ − (*E_F_*/Γ_*E*_)^*p* − 1^. A broader Gaussian DOS results in a smaller Seebeck coefficient while a value of *p* closer to 1 produces a flatter *α* curve. The point *E_F_* = 0 where *α* vanishes coincides with 50% doping. Intrinsic disorder plays a complementary role and dictates an upper bound on the trade‐off curve—larger Γ_*i*_ depresses the *α* even at low doping and flattens the curve, typically from *α*∝*σ* ^−1/2.5^ to *σ* ^−1/4^.

Taking the dopant‐induced Γ_*E*_ while fixing the shape parameter to different values, however, produced a significant difference in the *α* versus *σ* curve, whose slope decreased with *p* (black lines in Figure 2b and Figure S2b, Supporting Information). The difference is largest at low‐to‐moderate doping when carriers are predominantly in the tail of the DOS. In the presence of the heavy tail, doping moved *E_F_* closer to the center of the DOS while the transport energy *E_T_* = 〈*E_i_* 〉 initially decreased as the trap‐like states in the tail, which do not contribute significantly to transport, are filled first. This results in a lower *α*∝*E_F_* − *E_T_*, decreasing the Seebeck along with the conductivity. The *α* versus *σ* curve with the DOS computed from Equation (5), where both Γ_*E*_ and *p* are varying with doping (solid line in Figure 2b), scales as *α*∝*σ* ^−1/8^, in close agreement with data from our experiments.

Previous works have developed empirical relationships between *α* and *σ* that scale as^[^ ^15^, ^16^ ^]^ *α*∝ln *σ*,  *α*∝*σ* ^−1/4^, and more generally as *α*∝*σ* ^−1/*s*^ in the Kang–Snyder model,^[^ ^8^ ^]^ where *s* is the transport parameter, without establishing a connection to a specific material property. Here we have connected the transport parameter *s* to the shape of the DOS as it evolves in the presence of dopant‐induced energetic disorder. We find that polymers that retain a more Gaussian DOS, stemming from a higher *ε*, larger on‐site energy, or a more homogenous distribution of dopants, exhibit the *α*∝*σ* ^−1/*s*^ behaviour with *s* between 2.5 and 4, while polymers that encounter significant long‐range Coulomb interactions have *s* ranging from 6 to 8 (Figure S2c,d, Supporting Information). It is interesting to note that, at comparable values of Γ_*E*_, smaller *p* values have the effect of flattening the *α* versus *σ* curve (Figure S1b, Supporting Information), indicating that the ideal DOS for TE applications would be a sharp narrow Gaussian with an exponential tail (small p and Γ_*E*_). However, the presence of an exponential tail due to the dopants is always correlated with an increase in Γ_*E*_, indicating that the path forward is by mitigating the dopant‐induced disorder.

### 2.2 Experimental Validation

We hypothesized that the detrimental effect of dopant‐induced disorder on charge transport and the *α* versus *σ* curve can be mitigated by increasing the dielectric constant, which we have shown to counteract the coulombic broadening of the DOS and keep it nearly Gaussian (Figure 1). To test our hypothesis, we studied the impact of dielectric constant on the thermoelectric properties of P3HT, a well‐studied conjugated polymer for thermoelectric applications. The dielectric constant of a polymer can be changed by two methods: 1) without altering the chemical structure of the polymer by blending the polymer with additives (or “fillers”) or 2) by altering the chemical structure of the polymer by appending polar groups to the side chains^[^ ^17^ ^]^ or the backbone.^[^ ^18^, ^19^, ^20^ ^]^ For our study, we chose to prepare composites of conjugated polymers with colloidal nanocrystals of dielectric perovskite oxides with different dielectric constants. This method is straightforward and can be deployed to vary the dielectric constant of the conjugated polymer of interest by simply using a different dielectric additive.

**Figure 3:**
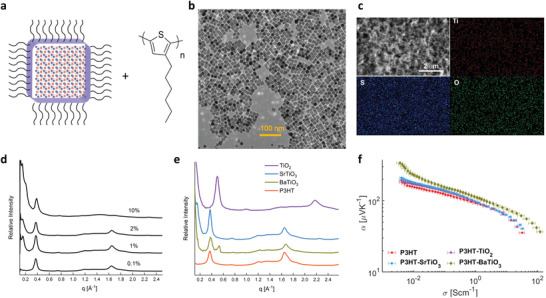
Fabricating polymer‐nanocrystal composites for enhancing thermoelectric performance and its morphological characterization. a) Scheme of components used to fabricate the polymer composites. b) TEM image of BaTiO_3_ nanocrystals shows consistent size and cubic shape. c) SEM‐EDS image shows a uniform distribution of BaTiO_3_ nanoparticles in the polymer composite film. The signal is weak due to the low concentration and smaller size of the nanoparticles. See also Figure S4c, Supporting Information. d) In‐plane X‐ray scattering pattern of SrTiO_3_‐P3HT composites with different ratio of SrTiO_3_ nanocrystals to P3HT. The disappearance of the (020) peak at SrTiO_3_‐P3HT composition ratios of 10% (wt./wt.) delineates a concentration limit for adding nanocrystals without affecting the polymer nanostructure. e) In‐plane X‐ray scattering pattern of SrTiO_3_ and BaTiO_3_ composites shows no change in the signature peaks of P3HT. TiO_2_ composite shows the formation of polymorph form II of P3HT. f) Plot of *α* versus *σ* experimental values of BaTiO_3_, SrTiO_3_, and TiO_2_‐P3HT composite and pristine P3HT. SrTiO_3_ and TiO_2_ composites showed no change in the *α* and *σ* interplay in comparison to pristine P3HT. The error horizontal bars represent the error in the electrical conductivity due to the variation in film thicknesses of thick films.

We synthesized colloidal nanocrystals of TiO_2_, SrTiO_3_, and BaTiO_3_ using established protocols. These nanocrystals were ≈10 nm in size and have a narrow size distribution, as characterized with transmission electron microscopy (TEM) and X‐ray diffraction. **Figure** **3**b shows a TEM image of BaTiO_3_ nanocrystals showing almost identical cubic shape and size. The nanocrystals are capped with oleic acid as ligands, which increases the miscibility between the additive and the polymer. We then fabricated polymer composite films by drop casting of a solution of nanocrystals and the polymer. The thicknesses of the composite films are about 4 µm, as characterized with profilometry. To ensure an adequate dispersion of the nanocrystals within the polymer films, we image the elemental composition of the films with scanning electron microscopy with energy dispersive spectroscopy (SEM‐EDS). As seen in Figure 3c, a BaTiO_3_ image exhibits a uniform distribution of Ti and O signals, indicating an adequate dispersion of the nanocrystals across the polymer film.

We used X‐ray scattering to understand the impact of incorporating nanocrystals on the polymer morphology. We used peaks associated with lamellar (100) and *π*–*π* stacking (020) of P3HT. We observed no significant change in the scattering patterns and peak positions of these peaks in P3HT composites containing 0.1% to 2% of SrTiO_3_ and BaTiO_3_ nanocrystals_._ These results indicate that the nanocrystal additives do not interfere with crystalline packing and may be present in the amorphous domains of the polymer. However, we did observe a shift in peaks to higher *q* values in P3HT‐TiO_2_ composites, which matches with P3HT polymorph form II.^[^ ^21^, ^22^ ^]^

We then probed the effect of nanocrystal concentration on the polymer morphology. We fabricated composites with different concentration of SrTiO_3_ nanocrystals and evaluated the polymer morphology with wide‐angle X‐ray scattering (WAXS). As seen in Figure 3d, the in‐plane WAXS pattern shows the disappearance of the (020) peak at 10% (wt./wt.) SrTiO_3_‐P3HT. This result establishes an upper boundary for fabricating polymer‐nanocrystal composites without affecting the crystalline regions of P3HT. We also evaluated the out‐of‐plane scattering pattern with grazing‐incidence wide angle X‐ray scattering (GIWAXS) and show the presence of same signature crystalline peaks for a 2% BaTiO_3_‐P3HT polymer composite and for the pristine polymer. These data indicate that at lower composition ratios, the BaTiO_3_ and SrTiO_3_ nanocrystals do not interfere with the crystal packing or orientation of the crystalline domains of P3HT. Based on these data, we concluded that at and below 2% (wt./wt.), the nanocrystals may be present in the amorphous domains of the polymer.

To evaluate the thermoelectric properties and obtain the *α*–*σ* trade‐off curve experimentally, we used a dedoping method with I_2_ that we have we have reported previously,^[^ ^3^, ^5^ ^]^ described in detail in the Experimental section. Our method captures the trend of *σ* and *α* over a four‐orders of magnitude *σ* window using a single sample and without modulation doping as the polymer gradually dedopes over time. Our method also has two significant advantages over existing methods: 1) it avoids any interfacial effects on the DOS that arise from modulation doping using field‐effect transistors; and 2) the overall polymer film morphology is essentially maintained during the measurement over a broad range of carrier concentration. As shown in Figure 3f, the log–log plot exhibits an upward‐right shift of the *α* versus *σ* curve for BaTiO_3_ composites in comparison to the pristine polymer, indicating a simultaneous improvement in both *α* and *σ*. The *α* versus *σ* curve for composites with lower *ϵ* additives (TiO_2_ or SrTiO_3_) did not show any significant change from pristine P3HT (Figure 3f), particularly at high dopant concentrations. To explore the effect of higher concentration of nanocrystals on the thermoelectric properties, we prepared a 50% (wt./wt.) SrTiO_3_‐P3HT composite. The *α*–*σ* trade‐off curve shows suppression of both *α* and *σ* (Figure S10, Supporting Information). For films with higher nanocrystal content, we were unable to measure the *α* versus *σ* curve because of extremely low *σ*. These findings are consistent with our expectations that nanocrystals are insulating and thus do not contribute to the charge transport pathways.

We measured the dielectric permittivity of the polymer‐nanocrystal composite films with electrochemical impedance spectroscopy (EIS), which obtains the bulk dielectric permittivity (*ϵ*_bulk_) of a composite material across a frequency range. As can be seen in the Figure S6, Supporting Information, at 1 kHz, BaTiO_3_ composites exhibits a significant increase in *ϵ*_bulk_ from 3.5(±0.1) to 84.6(±1.4) when compared to the pristine polymer, whereas SrTiO_3_ nanocrystals showed a *ϵ*_bulk_ of 14.0(±3.6). This dramatic enhancement arises from the polarization induced by BaTiO_3_, which is consistent with previous work on similar polymer‐nanocrystal composites.^[^ ^23^ ^]^ The measured *ϵ*_bulk_ value for P3HT matches very well with the value obtained with the numerical simulations' fit.

### 2.3 Analysis of Experimental Results and the Role of Energetic Disorder

**Figure 4:**
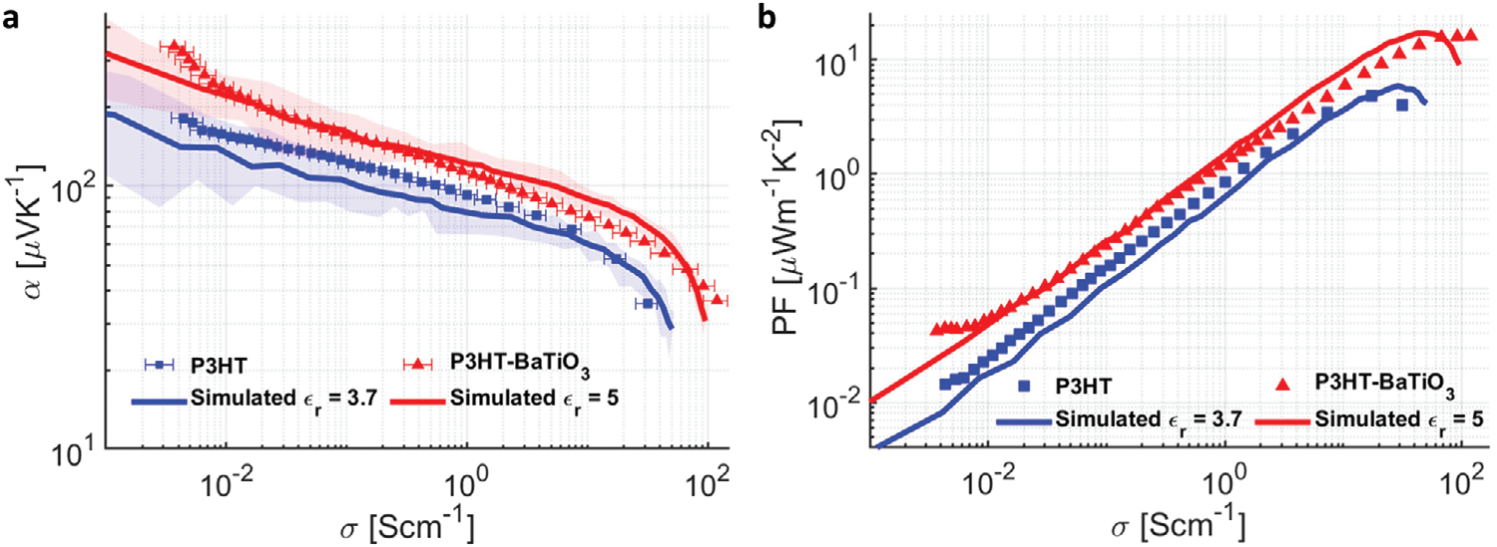
Higher dielectric permittivity leads to a higher power factor in OSCs. a) Plot of simulated *α* versus *σ* values and experimental values of BaTiO3‐P3HT composite and pristine P3HT. BaTiO_3_‐P3HT composite shows a fit to *ε*_*r*_ = 5 while pristine P3HT fits *ε*_*r*_= 3.7. The simulations were iterated 25 times; the solid lines represent averaged values and the shaded region represents the minimum and maximum values. The horizontal error bars represent the error in the experimental electrical conductivity due to the variation in film thicknesses. b) Power factor as a function of carrier density. There is an approximately fourfold increase in power factor from 4.8 to 16.2 as *ε*_*r*_ is increased.

To understand the experimental trends, we fit the experimental data with our simulations and found that the *α* versus *σ* curve for pristine P3HT can be fit with *ε*_*r*_ = 3.7 whereas BaTiO_3_‐P3HT composite with *ε*_*r*_ = 5, shown in **Figure** **4**a. This is consistent with our expectation that a higher dielectric permittivity will counteract the effect of dopant‐induced Coulomb interactions on the polymer DOS, leading to better charge transport properties. The simultaneous increase in the Seebeck coefficient and conductivity results in a tremendous increase in the thermoelectric power factor (PF), given by *PF* = *α* ^2^ *σ*, as shown in Figure 4b. With *ε*_*r*_=3.7, we observed a peak PF of 4.8 μWm^−1^ K^−1^ in pristine P3HT, which increased to 16.2 μWm^−1^ K^−1^ with the addition of BaTiO_3_. However, we recognize that there is a discrepancy between the measured *ϵ*_bulk_ and the value needed to fit the curve for the BaTiO_3_ composites. We posit that the value needed to fit the curve may be an effective dielectric permittivity (*ϵ*_ef_) experienced by the electric fields between charge carriers and dopant ions, which depends on their average distance from the nanocrystal. The dopant‐induced energetic disorder is primarily caused by the Coulomb interaction with the nearest dopant, whose average distance can be estimated from $N_{d}^{-1/3}$ to span 1–10 nm in our experiments and calculations and is therefore smaller than the average size of the nanocrystals (10 nm). We surmise that, to increase the dielectric permittivity experienced by the electric field between charge‐carriers and dopant ions, we need to use nanocrystals with high *ε*_r_ or to tailor the ligands on the nanocrystals so that they place closer to the radical cation on the polymer or its counterion. Nonetheless, applying such a strategy could compromise the polymer morphology, which can affect charge transport.

**Figure 5:**
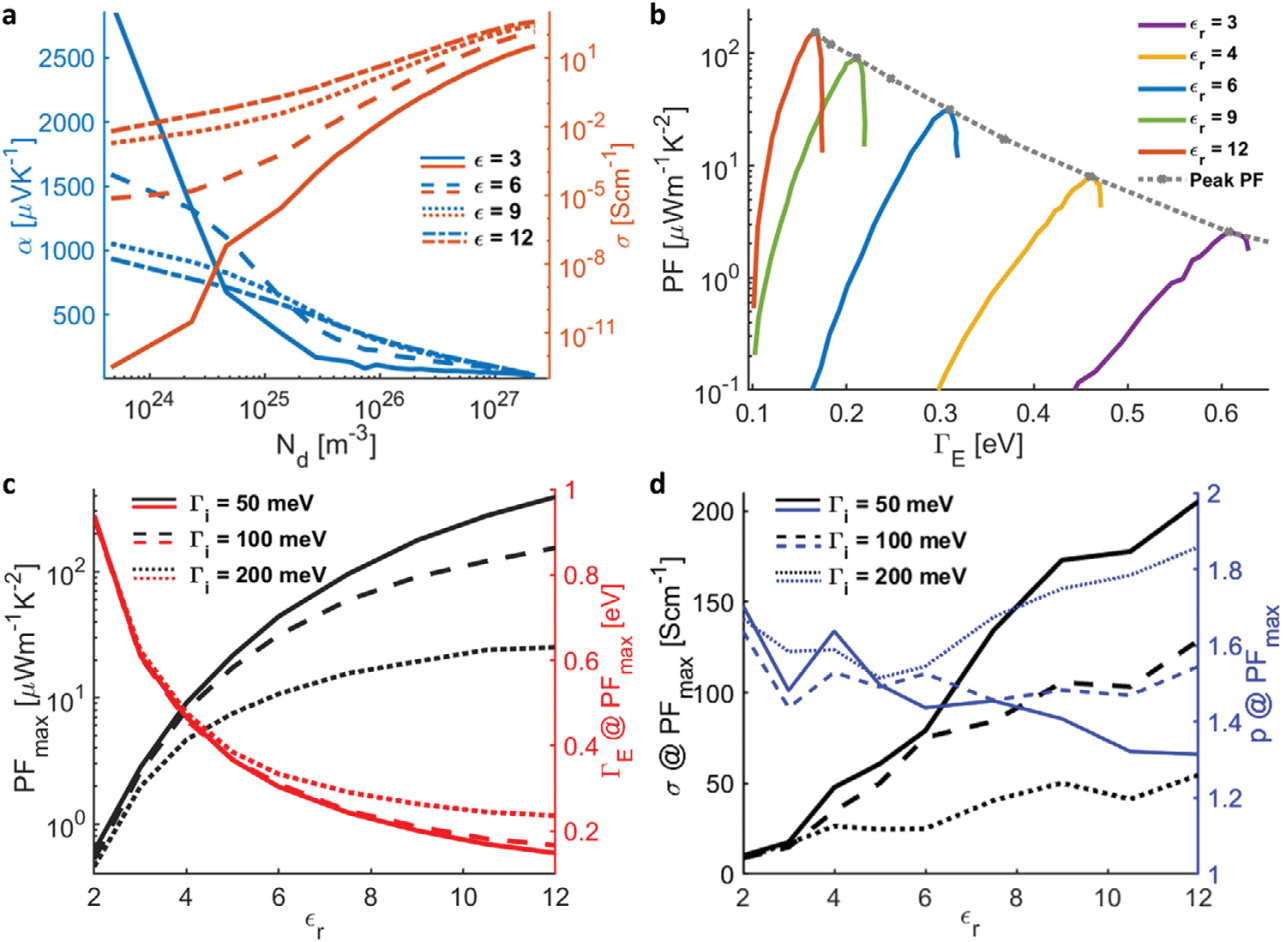
Power factor enhancement and the role of energetic disorder. a) *α* and *σ* as a function of carrier density showing the respective increase with increasing *ϵ* at medium to high doping concentrations. b) Power factor versus energetic disorder showing that increasing the dielectric constant reduces the energetic disorder at all doping concentrations and leads to higher power factor. c) Maximum power factor and the energetic disorder versus *ε*_*r*_, showing that gains in the power factor saturate beyond *ε*_*r*_>12, when they are limited by intrinsic disorder. d) Conductivity *σ* and shape parameter *p* as a function of *ε*_*r*_, plotted at the doping concentration corresponding to maximum power factor, showing increasing conductivity with decreasing intrinsic disorder, particularly when low intrinsic disorder and small *p* value produce a narrow main DOS with a heavy tail.

We examine conductivity and Seebeck versus doping in **Figure** **5**a. Increasing *ε*_*r*_ mitigates dopant‐induced disorder and produces a more sharply peaked DOS, boosting Seebeck at high doping due to the increased separation between the transport energy and the Fermi level (Figure S7a, Supporting Information). The impact of dielectric screening on conductivity is even more dramatic—carriers in a heavy‐tailed DOS get “stuck” in the trap‐like states deep in the tail. Transport improves at higher doping concentrations when the tail states are filled, discerned by the steeper conductivity curves in Figure 5a. Conductivity increases super‐linearly with doping,^[^ ^24^ ^]^ following a power‐law^[^ ^25^ ^]^ trend $\sigma \propto N_{d}^{\zeta }$, with the average exponent related to disorder *ζ* ∝ Γ_*E*_ (see Figure S7b, Supporting Information). A narrower DOS reduces the difference Δ*E_ij_* between energies of neighboring sites, which increases the probability of favorable hopping pathways by alleviating the required thermal assistance by absorption of phonons, resulting in a much higher conductivity for the higher *ε*_*r*_ case. While both Seebeck and conductivity depend on the complex interplay between doping and energetic disorder, the peaks in the PF exhibit an inverse trend with energetic disorder, shown in Figure 5b. Increasing the *ε*_*r*_ from 3 to 12 mitigates dopant‐induced energetic disorder, pushing the curves to lower Γ_*E*_ while increasing the height of the peak in the PF. Doping is more effective at higher *ε*_*r*_ as carriers contribute more readily to transport in the absence of the deep coulombic tail. Consequently, we achieve higher power factors at lower doping concentrations (Figure S8, Supporting Information). While there is a modest increase in Seebeck with higher *ε*_*r*_ at a fixed doping concentration, reaching the peak PF requires less doping, which effectively raises the Seebeck at the peak (Figure S9a, Supporting Information). We note that a recent paper observed a quadratic relationship between power factor and dielectric constant in crystalline inorganic thermoelectrics.^[^ ^26^ ^]^

However, the maximum attainable PF saturates for *ε*_*r*_ > 12. Beyond this value, the polymer's intrinsic energetic disorder acts as a limiting factor in the highest attainable PF. Dielectric screening can only mitigate the broadening of the DOS by carrier‐dopant Coulomb interactions but not the intrinsic disorder within the polymer, which is related to structural disorder and depends on the polymer's morphology.^[^ ^27^ ^]^ In Figure 5c, we plot the peak PF (left) and the energetic disorder (right), both as functions of the dielectric permittivity, for several values of intrinsic disorder Γ_*i*_ (50, 100, and 200 meV), echoing the inverse relationship between peak PF and Γ_*E*_. The *p* value plays a complimentary role (Figure 5d) as smaller intrinsic disorder makes the DOS tail more pronounced, leading to smaller *p*. There is a synergy between screening and morphology—when dopant‐induced disorder is minimized by dielectric screening, conductivity, shown by black lines in Figure 5d, becomes inversely proportional to the remaining intrinsic disorder. At lower intrinsic disorder, the PF_max_ reached 391 μWm^−1^ K^−2^, which corresponds to a TE figure‐of‐merit zT = *α* ^2^ *σT*/*κ* of 0.6 at room temperature (RT), assuming a typical thermal conductivity *κ* ≈ 0.2 Wm^−1^ K^−1^.^[^ ^28^ ^]^ However, this was calculated with the same parameters we used to fit the P3HT measured data and further increases may be achievable in other polymers. In order to estimate the highest TE figure‐of‐merit that could be reached with a dielectric constant of 12, we explore the impact of other simulation parameters, namely the intrinsic disorder Γ_*i*_, overlap *γ*, and dopant radius *R*_S_. Changing parameters in the simulation to values within the range encountered in polymers, additional improvements with dielectric screening were observed (Figure S9b, Supporting Information) and the PF reached 2170 μWm^−1^ K^−1^ at *ε*_*r*_ = 12, which would correspond to a RT zT of 3.2. However, increasing the effective dielectric constant to 12 throughout a polymer remains a formidable future challenge.

## 3 Conclusion

We conclude that increasing dielectric screening can mitigate dopant‐induced traps and have a positive impact on the transport properties of doped polymers with an intrinsically low *ϵ*. At the same time, we develop an experimental framework that can alter the permittivity of the material without affecting its intrinsic properties, BaTiO_3_‐induced dielectric screening can decrease Coulomb interactions and thus the magnitude of the heavy‐tailed DOS. This synergetic computational and experimental study opens avenues towards developing more effective strategies to use dielectric screening for mitigating the effect of dopants in the DOS. Our results indicate that polymers with high dielectric permittivity are a fertile new avenue of research in organic TEs and a path forward to obtain zT values well over the highest reported 0.4 so far.^[^ ^29^ ^]^ Beyond improving TE performance, we note that most of the improvement we observed in the PF comes from increases in the conductivity, particularly at low to medium doping concentrations, which is broadly useful in organic electronics. Long‐range Coulomb interactions also impact other systems such as photogenerated carriers in organic photovoltaics. Raising the dielectric permittivity of the active layer could increase exciton dissociation, enhancing photovoltaic performance, and improve carrier mobility in field effect transistors.

## 4 Experimental Section

### Hopping Transport Simulation

We calculate *α* and *σ* by numerically solving the PME that describes phonon‐assisted carrier hopping between localized sites whose energies are sampled from the carrier DOS. The hopping rate between sites (*i*–*j*) is calculated from the Miller–Abrahams rate equation^[^ ^30^ ^]^ $W_{ij}=\nu_{0}\exp\left(-2\gamma_{ij}R_{ij}\right)\left[N\left(\Delta E_{ij}\right)+\frac{1}{2}\pm \frac{1}{2}\right]$, where *ν*_0_ = 5 × 10^12^ s^−1^ is the attempt to escape frequency, *γ* = 0.75 is the overlap factor (*γ_ij_* = *γ_i_* +*γ_j_*, *γ_i_*, and *γ_j_* are the site‐specific contributions obtained from a Gaussian distribution of width Σ_*ij*_ = *γ*/4 and *R_ij_* is the distance between the sites. *N*(*E*) is the Bose–Einstein distribution with $+\frac{1}{2}$ for hops upwards in energy (*E_i_* > *E_j_*) by absorption of a phonon and $-\frac{1}{2}$ for downward hops with the emission of a phonon. Δ*E_ij_* = *E_j_* − *E_i_* − *eF*Δ*R*_*ij*,*x*_ where, *E_i_* and *E*_j_ are the energies of the sites and *F* = 10^6^ Vm^−1^ is the externally applied electric field.^[^ ^3^ ^]^ These are the values used throughout the simulation.

We numerically solve the PME to compute the time‐averaged occupational probabilities of the sites using a non‐linear iterative solver and the initial site occupation probability is given by the Fermi–Dirac distribution. In steady‐state, $\frac{dp_{i}}{dt}=0=\Sigma_{j}\left[W_{ij}p_{i}\left(1-p_{j}\right)-W_{ji}p_{j}\left(1-p_{i}\right)\right]$ where *p_i_* is the occupation probability of a site *i* and *W_ij_* is the hopping transition rate, and the whole term is summed over the neighbor sites *j*.^[^ ^31^ ^]^ The current density *J* is found by a summation over all the carriers in the direction of the applied field, $J=\frac{e}{a^{3}N}\sum_{i,j}W_{ij}p_{i}\left(1-p_{j}\right)R_{ij,x}$ and the Seebeck coefficient is calculated as $S=\frac{E_{F}-E_{T}}{eT}$ where, *E*_T_ is the average transport energy, calculated from $E_{T}=\left\langle E_{i}\right\rangle =\frac{\sum_{i,j}E_{i}W_{ij}p_{i}\left(1-p_{j}\right)R_{i,j,x}}{\sum_{i,j}W_{ij}p_{i}\left(1-p_{j}\right)R_{i,j,x}}$.^[^ ^32^ ^]^ We simulate a 35 × 35 × 50 lattice of sites with an average distance between adjacent sites *a* = 0.6 nm, and consider up to the fifth‐nearest neighbor.

### Solving the Non‐linear PME

We solve the non‐linear PME using a standard iterative non‐linear solver. First, we cast the PME as zero‐finding for a system of equations $F_{i}\left(p\right)=\sum_{j}\left[W_{ij}p_{i}\left(1-p_{j}\right)-W_{ji}p_{j}\left(1-p_{i}\right)\right]=0$, which can be written in terms of the in‐ and out‐scattering as *F_i_* (*p*) = *p_i_S*_out_(*p*) − (1 − *p_i_*)*S*_in_(*p*), where $S_{\mathrm{out}}\left(p\right)=\sum_{j}\left[W_{ij}\left(1-p_{j}\right)\right]$ and $S_{in}\left(p\right)=\sum_{j}\left[W_{ji}p_{j}\right]$. Since both in‐ and out‐scattering terms depend on the unknown *p*, *F_i_* (*p*) is nonlinear and a fixed‐point iteration can stall, resulting in poor convergence for some cases. Hence, we follow a fixed‐point iteration for the *p_i_* such that $p_{i}^{n+1}=S_{\mathrm{in}}\left(p^{n}\right)/\left[S_{\mathrm{in}}\left(p^{n}\right)+S_{\mathrm{out}}\left(p^{n}\right)\right]$ with the initial $p_{i}^{0}$ being the Fermi–Dirac distribution, only for the first few iterations and then use the resulting estimate of *p_i_* as an initial guess where we numerically solve for *F_i_* (*p*). Rather than solving for the site occupancies *p_i_*, we solve for their deviation away from equilibrium $\Delta p_{i}=p_{i}-p_{i}^{0}$. Combining this with the detailed balance condition $0=\Sigma_{j}\left[W_{ij}p_{i}^{0}\left(1-p_{j}^{0}\right)-W_{ji}p_{j}^{0}\left(1-p_{i}^{0}\right)\right]$, we get $F_{i}\left(p\right)=\Delta p_{i}S_{\mathrm{out}}\left(p\right)-\left(1-p_{i}^{0}\right)S_{\mathrm{in}}\left(p\right)=0.$

We arrange the 35 × 35 × 50 array of Δ*p_i_*’s into a column vector *p* and compute the Jacobian matrix of derivatives of *F_i_* with respect to *p_j_* as *J_ij_* = *dF_i_*/*dP_j_* = −*W_ji_* (1 − *p_i_*). Then we apply the Levenberg–Marquardt algorithm,^[^ ^33^ ^]^ as implemented in MATLAB's fsolve function, with the known Jacobian matrix, which requires a linear solve at each iteration but typically converges in a few iterations due to its high rate of convergence. The linear solver is a preconditioned conjugate gradients algorithm with a banded preconditioner based on an incomplete Cholesky factorization.

### Density‐of‐States Model Including Carrier‐Dopant Electrostatic Interactions

Arkhipov et al.,^[^ ^4^ ^]^ have shown that Coulomb interactions between carriers and ionized dopants result in a heavy‐tailed DOS given by $g\left(E\right)=\frac{4\pi q^{6}N_{d}}{{\left(4\pi \varepsilon_{0}\varepsilon \right)}^{3}}\int_{-\infty }^{0}\frac{dE_{c}}{E_{c}^{4}}\exp\left[\frac{4\pi N_{d}}{3}\frac{q^{6}}{{\left(4\pi \varepsilon_{0}\varepsilon E_{c}\right)}^{3}}\right]g_{i}\left(E-E_{c}\right)$, where *N*_d_ is the dopant concentration, *E*_c_ is the potential energy of the Coulomb interaction, and *g*_i_ is the intrinsic Gaussian DOS centered at 0 energy and given by $g_{i}\left(E\right)=\frac{N_{i}}{2\pi \Gamma_{i}^{2}}\exp\left(-\frac{E^{2}}{2\Gamma_{i}^{2}}\right)$ where *N*_i_ is the intrinsic concentration. However, *n* the presence of dopant clustering, the probability density *w(r)* of the minimum distance at which the nearest dopant cluster is present is given by a Poisson distribution $w\left(r\right)=4\pi r^{2}N_{s}\exp\left(\frac{4\pi }{3}N_{s}r^{3}\right),\;$where *N*_s_ = *N*_d_/*C*_s_ is the density of clusters and *C*_s_ is the number of dopants in each cluster. The potential energy of the Coulomb interaction between the localized charge carrier and dopant cluster is now *E*_c_(*r*) =   − *C*_s_ *q* ^2^/(4*πε*_0_ *ε* *r*). Combining these equations to obtain the energy distribution of localized states over the intrinsic distribution *g*_i_ and energy *E*_c_ we have:^[^ ^4^, ^5^ ^]^

(2) $$g\left(E\right)=\frac{4\pi q^{6}N_{s}C_{s}^{3}}{{\left(4\pi \varepsilon_{0}\varepsilon \right)}^{3}}\int_{-\infty }^{0}\frac{dE_{c}}{E_{c}^{4}}\exp\left[\frac{4\pi N_{s}C_{s}^{3}}{3}\frac{q^{6}}{{\left(4\pi \varepsilon_{0}\varepsilon E_{c}\right)}^{3}}\right]g_{i}\left(E-E_{c}\right)$$

For the contributions arising from energies satisfying *E*_c_ ≫ Γ_*E*_, which correspond to instances where carriers are close to the ionized dopants, the integral in Equation (2) can be further simplified. This condition is primarily satisfied by states in the heavy tail of the DOS, representing deep traps. The intrinsic DOS can then be approximated by a delta function *g*_i_(*E* − *E*_c_) ≈ *N_i_δ*(*E* − *E*_c_) so that the whole integral can be evaluated analytically

(3) $$\begin{array}{ccc} g_{\mathrm{tail}}\left(E\right) & = & 4\pi E_{\mathrm{Coulomb}}^{3}\int_{-\infty }^{0}\frac{dE_{c}}{E_{c}^{4}}\exp\left[\frac{4\pi }{3}\frac{E_{\mathrm{Coulomb}}^{3}}{{E_{c}}^{3}}\right]\\ N_{i}\delta \left(E-E_{c}\right) & = & 4\pi N_{i}\frac{E_{\mathrm{Coulomb}}^{3}}{E^{4}}\exp\left[\frac{4\pi }{3}\frac{E_{\mathrm{Coulomb}}^{3}}{E^{3}}\right] \end{array}$$

where the pre‐factor groups together all the constants into:

(4) $$E_{\mathrm{Coulomb}}=\frac{q^{2}C_{s}}{4\pi \varepsilon_{0}\varepsilon }N_{s}^{1/3}$$

which is the average Coulomb energy of interaction between two dopants. This heavy tail of the DOS exhibits a combination of exponential and power‐law dependence on energy, departing from the intrinsic Gaussian shape. The *E* ^4^ term in the denominator of the DOS gives the tail a polynomial shape when doping is low and the exponential term is close to 1.

However, the model in Equation (2) produces a tail with very deep traps because it allows *E*_C_(*r*) to diverge to − ∞ as *r* → 0, equivalent to treating dopants as point charges. Such infinitely deep traps have a dramatic impact on conductivity that has been noted in the literature^[^ ^4^, ^34^ ^]^ and resolved by limiting the most negative value of *E*_c_(*r*), and thus the lower limit of the integral in Equation (2), to the on‐site energy of the dopant, typically −0.5 to −1 eV.^[^ ^34^ ^]^ Doing so is equivalent to limiting the distance to the nearest dopant to be no smaller than a dopant radius *R*_S_ obtained by setting *E*_c_(*R*_S_) equal to the on‐site energy, with the radius of 4–8 Å, corresponding to on‐site energy of −0.5 to −1 eV. More generally, a finite‐sized dopant can be modelled by a charge distribution instead of a point charge. For a Gaussian charge distribution, the potential energy becomes $E_{c}\left(r\right)=\;-\frac{C_{s}q^{2}}{4\pi \varepsilon_{0}\varepsilon \;r}\mathrm{erf}\left(\frac{r}{R_{s}}\right)$. Then the integral for the DOS must be performed with respect to nearest dopant distance *r* rather than energy because *E*_c_(*r*) is no longer invertible, resulting in:

(5) $$g\left(E\right)=4\pi N_{s}\int_{0}^{\infty }r^{2}\exp\left(\frac{4\pi }{3}N_{s}r^{3}\right)g_{i}\left[E-E_{C}\left(r\right)\right]dr\;$$

This approach is also useful in capturing the finite size of dopant clusters, which can be assigned a radius *R*_S_ instead of being treated as being point charges. Other formulations are possible, such as treating the ionized dopant's charge distribution as a shell of radius *R*_S_, in which case the Coulomb potential inside *r* < *R*_S_ becomes constant $E_{c}\left(r\right)=-\frac{C_{s}q^{2}}{4\pi \varepsilon_{0}\varepsilon \;R_{S}}$; coincidentally, this is also the maximum value reached by the potential from a Gaussian charge distribution.

We have implemented the above and found the Gaussian distribution to produce the smoothest DOS tail, while the choice of dopant size/radius has far more impact on the DOS than how the dopant distribution is modelled. We compute the DOS for a given doping concentration and cluster size by numerical quadrature of Equation (5), after breaking it up into two intervals, *r* < *a* and *r* > *a*, and normalizing as described by Zuo et al.^[^ ^34^ ^]^ We use Γ_*i*_ = 100 meV and *ε*_*r*_ = 3.7, unless noted otherwise, and *r* = 2 Å in our simulations which would correspond to on‐site energy of −1.9 eV. Next, we use the rejection sampling technique to generate an energy distribution that follows from the calculated DOS, and an energy value is randomly assigned to each site from the resulting distribution. We then use the bisection method to iteratively find the corresponding Fermi level *E*_F_ for the given carrier density. The iteration typically converges to sufficient precision within 20 iterations. We iterate the entire simulation at each dopant concentration 25 times, to reduce the sampling error from the randomly assigned site energies.

### Polymers

P3HT (*M*_w_: 36 kDa, regioregularity: 96% HT) was purchased from Rieke Metals. Iodine crystals were purchased from Sigma Aldrich. All solvents were purchased from commercial vendors.

### Film Preparation

P3HT was dissolved in chloroform to prepare 10 mg mL^−1^ solutions by stirring and heating at 45 °C for at least 2 h. 1.1 × 2.2 cm glass slides were hand cut for thermoelectric measurements, 1.5 × 1.5 cm half ITO‐covered glass slides used for dielectric measurements, and 1.5 × 1.5 cm p‐doped silicon substrates for SEM‐EDS imaging.

All substrates were sonicated with soap/water, water, acetone, and isopropanol for 20 min each and dried in an oven at 130 °C. The substrates were cleaned under ozone (UVO Cleaner, Model 342, Jelight Company, Inc.) for 10 min. All films were prepared by drop casting the solutions in preheated slides at 45 °C. The films were left under vacuum (>10^−2^ mbar) for at least 24 h to evaporate residual solvents. The thickness of the films (≈4 µm) was measured with a profilometer at three different points across the film.

### Strontium Titanate Nanocrystals

Synthesis of strontium titanate nanocrystals was carried out using a hydrothermal method. In a typical synthesis, 1.25 mmol of each of bis(ammonium lactate) titanium dihydroxide (TALH) and strontium hydroxide (Sr(OH)_2_) were dissolved with 30 mL of distilled water in a 45 mL Teflon‐lined autoclave. The pH of the solution was then adjusted to 12.1 with a 10 m tetramethylammonium hydroxide (NMe_4_OH) solution followed by the addition of oleic acid (2.5 mmol). The reaction vessel was then sealed and heated to 200 °C in oven for 24 h. The resulting nanocrystals were collected, washed with ethanol three times, and suspended in non‐polar solvents.

### Barium Titanate Nanocrystals

Synthesis of barium titanate colloidal nanocrystals was also carried out by a similar hydrothermal method. In a typical preparation, 1.5 mmol of each TALH and Ba(OH)_2_ were dissolved in 24 mL distilled water followed by addition of 6 mL of sodium hydroxide (NaOH, 5 m). The reaction solution was then transferred to a 45 mL Teflon‐lined autoclave and oleylamine (6 mmol) and oleic acid (6 mmol) were added. The sealed autoclave was placed in custom‐made aluminum block housing that was heated to 215 °C and stirred constantly for 24 h using a stirring hotplate. After the synthesis, autoclave was cooled to room temperature and the solid product was collected, washed with ethanol several times, and then dissolved in nonpolar solvents.

### Titanium Dioxide Nanocrystals

Synthesis of TiO_2_ nanocrystals was carried out by a solvothermal method. In a typical preparation, 1.5 mmol of titanium butoxide were mixed with 7.5 mmol of oleic acid and 7.5 mmol of oleylamine in 1.1 mL of ethanol. The obtained mixture was then transferred to a 45 mL Teflon‐lined autoclave containing 5.1 mL of 96% ethanol in water (v/v) and heated to 200 °C for 18 h. After the synthesis, the autoclave was cooled down to room temperature and the solid product was collected, washed with ethanol several times and resuspended in non‐polar solvents to produce colloidal solutions.

### Nanocrystal Characterization

The solution concentration of nanocrystals was calculated with ICP‐OES. The size of the nanocrystals was determined by TEM and powder x‐ray diffraction patterns. To prepare the polymer composites, weight ratios of nanocrystal solutions to polymer solutions were used. The solution blends were drop casted by following the same procedure as for the pristine polymer.

### I_2_ Doping and Thermoelectric Properties

The I_2_ method was adapted from a previous work.^[^ ^3^, ^5^ ^]^ The method consisted of transferring 50 mg of I_2_ into a 1 mL vial and placing this vial inside a larger glass container with the film inside. The films were doped at 75 °C for 2 h in sealed chamber with 50 mg of I_2_. After removing the film from the doping system, the instability of iodine caused the polymer film to dedope over time. Immediately, the polymer film was transferred into a custom‐built thermoelectric box to measure the electrical conductivity and the Seebeck coefficient as the dedoping proceeded. This electrically grounded box was equipped with two copper blocks: the temperature of the hot block was maintained with a heating element and the cold block was cooled with a water chiller. To create a temperature gradient, the films were placed on top of an insulating glass slide that was bridged between the two blocks. A PTFE block was used to hold four platinum probes in a four‐point probe arrangement and two k‐type thermocouples. To measure the electrical conductivity, a Keithley 2440 5A sourcemeter was used to source a bias of −0.1 to 0.1 V and generate an *I*–*V* curve. The conductance was calculated with the slope of this curve and normalized with the geometry of the film. A Keithley 2182A nanovoltmeter was used to measure the temperature‐induced voltage difference (∆*V*), and the thermocouple cables were used to monitor the temperature difference (∆*T*) in both sides of the film. The Seebeck coefficient was calculated by the empirical relationship $\alpha =\frac{\Delta V}{\Delta T}$.

### Dielectric Properties

To measure the dielectric properties, polymer‐nanocrystal solutions were dropcast onto an ITO slide. Aluminum electrodes (thickness: 500 nm, area: 6.44 mm^2^) were deposited with a shadow mask in a thermal evaporator to fabricate thin film capacitor devices. Electrochemical impedance spectroscopy (EIS) measurements were executed under inert conditions in a N_2_ filled glovebox with O_2_ and H_2_O levels below 0.1 ppm to determine the real (Z′) and imaginary (Z′′) impedance. The frequency scan was done in an open‐circuit correction configuration. The AC frequency was 100 Hz. The dielectric constant was determined by $\varepsilon^{\prime }=-\frac{d}{2\omega A\varepsilon_{0}\;}\cdot \frac{Z^{\prime \prime }}{{\left(Z^{\prime }\right)}^{2}+{\left(Z^{\prime \prime }\right)}^{2}}$, and the dielectric loss was determined by $\varepsilon^{\prime \prime }=\frac{d}{2\;\pi f\;A\;\varepsilon_{0}}\cdot \frac{Z^{\prime }}{{\left(Z^{\prime }\right)}^{2}+{\left(Z^{\prime \prime }\right)}^{2}}$ where Z′ is the real impedance, Z″ is the imaginary impedance, *f* is the frequency , *d* is the thickness of the film, *ε*_0_ is the vacuum dielectric constant, and *A* is the overlapping area between the electrodes.

### X‐Ray Scattering

WAXS measurements were performed in a SAXSLAB Ganesha 300XL X‐ray Scattering instrument. The instrument was equipped with a Xenocs GeniX 3D Cu K*α* source (*λ* = 0.15418 nm) and a Dectris Pilatus 30 K photon‐counting detector. The sample‐detector distance was ≈100 mm, and ultra‐high vacuum was applied to reduce background scattering. The films were peeled off from their substrates to prepare free‐standing thick films and perform in‐plane WAXS measurements.

### Scanning Electron Microscopy (SEM‐EDS)

Scanning electron microscopy (SEM) images were captured in a FEI (Thermo Fisher Scientific) Magellan 400 XHR‐SEM equipped with an Oxford X‐MAX 80 mm^2^ energy dispersive x‐ray spectrometer (EDS).

## Conflict of Interest

The authors declare no conflict of interest.

## Author Contributions

Z.A. and D.V. conceived the project and supervised the work. M.L.‐D. and S.S. carried out the experiments. Z.A. and M.U. developed the simulation code and M.U. ran the simulations. K.R.K. and M.A. synthesized the nanoparticles. M.U. and M.L.‐D. contributed equally to this work. All authors contributed to writing the manuscript.

## Supporting information

Supporting Information

## Data Availability

The data that support the findings of this study are available from the corresponding author upon reasonable request.
