# Supplementary material for: Raising Dielectric Permittivity Mitigates Dopant‐Induced Disorder in Conjugated Polymers
Source: Adv Sci (Weinh). 2021 Aug 11;8(19):2101087. doi: 10.1002/advs.202101087 (PMC8498903; doi:10.1002/advs.202101087)
Supplement: Supplementary file 1 — Supporting Information [file ADVS-8-2101087-s001.pdf]

© 2021 Wiley-VCH GmbH

ADVANCED SCIENCE
Open Access

Supporting Information

for *Adv. Sci.*, DOI: 10.1002/advs.202101087

Raising dielectric permittivity mitigates dopant-induced disorder in conjugated polymers

Meenakshi Upadhyaya,[1] Michael Lu-Díaz,[2] Subhayan Samanta,[2] Muhammad Abdullah,[2] Keith Dusoe,[3,4] Kevin R. Kittilstved,[2] Dhandapani Venkataraman,[2,#] Zlatan Akšamija[1,*]

Supplementary Information:

Raising dielectric permittivity mitigates dopant-induced disorder in conjugated polymers

Meenakshi Upadhyaya,[1] Michael Lu-Díaz,[2] Subhayan Samanta,[2] Muhammad Abdullah,[2] Keith Dusoe,[3,4] Kevin R. Kittilstved,[2] Dhandapani Venkataraman,[2,#] Zlatan Akšamija[1,*]

[1]*Electrical and Computer Engineering, University of Massachusetts Amherst*

[2]*Chemistry, University of Massachusetts Amherst*

[3]*Polymer Science and Engineering, University of Massachusetts Amherst*

[4]*Institute for Applied Life Sciences, University of Massachusetts Amherst*

Email: [*]zlatana@umass.edu, [#]dv@umass.edu

Impact of the DOS width (Γ_E) and shape parameter (p) on the α vs. σ curve

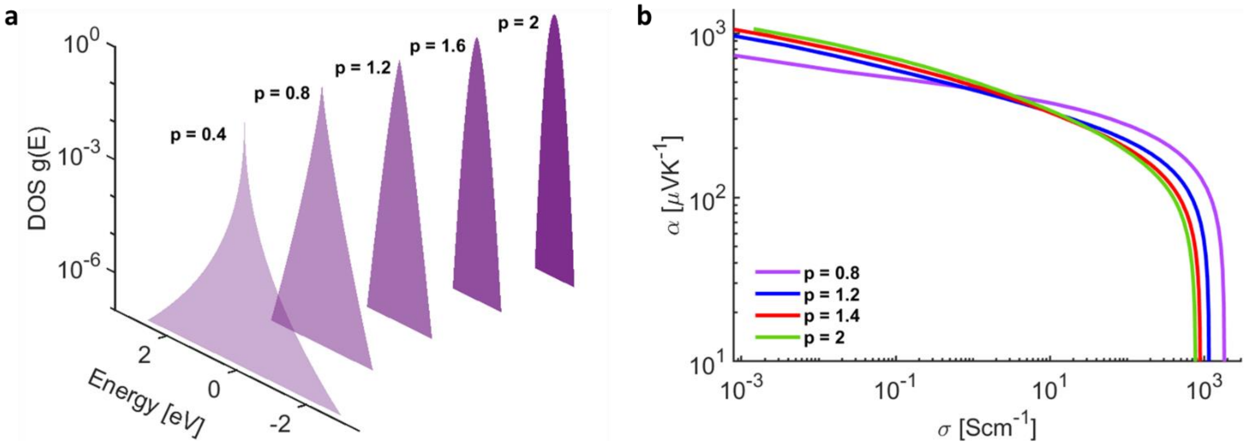

Supplementary Figure S1. a Generalized Gaussian distribution with shape parameter p values ranging from 0.4 to 2, showing the increasing heavy tail as p decreases. **b** The effect of shape parameter p on the α vs. σ curve with constant width $\Gamma_E = \Gamma_i = 100\ meV$. At constant width a lower p value has the effect of flattening the α vs. σ curve leading to a higher power factor.

Supplementary Note 1:

To precisely examine the slope of the α vs. σ curves across the entire range of doping concentration, we plot the running exponent $\beta = \frac{\mathrm{d}(\ln\alpha)}{\mathrm{d}\,(\ln\sigma)}$ vs. doping concentration. For a Gaussian DOS with constant width Γ_E across doping concentrations we find that the exponent remains constant for various Γ_E values (grey lines in Figure S2a). If we let Γ_E increase with doping concentration by extracting it from the DOS (Equation (5) in main text) but keep the shape Gaussian (p = 2), the average value of the exponent (in the flat region of the curve) increases to ~ -0.4 for Γ_i=100 meV and ~ -0.3 for Γ_i=200 meV due to the detrimental effect of Γ_E on α. If we take the dopant-induced Γ_E and fix the shape parameter to different values, we find that the lowest p value has the flattest curve (black lines in Figure S2b). The average exponent increases from ~ -0.1 to -0.4 as p goes from 0.8 to 2. For the blue and red lines in Figure S2b (standard case with DOS computed from Equation (5) in the main text), where Γ_E and shape parameter p are both changing for Γ_i values of 100 and 200 meV, we find that the exponent is close to ~ -0.1. Although the increase in Γ_E with doping has the net effect of increasing the slope of the α vs. σ curve the decrease in p value with doping has the opposite effect of flattening the curve.

Next, we fit the α vs. σ curves of P3HT and PDPP4T obtained from our previous work.[1] In our previous work we showed that different slopes of the α vs. σ curve stems from a different shape of the DOS due to the morphological difference in the distribution of dopants in the polymer. Polymers that retain a more Gaussian DOS due to homogenous distribution of dopants (PDPP4T doped at 75 ^{0} C) exhibit $\alpha \propto \sigma^{-1/4}$ behavior whereas polymers with a heterogenous distribution of dopants that have a more heavy-tailed DOS exhibit $\alpha \propto \sigma^{-1/s}$ with s between 6 and 8 (Figure S2c). Similarly, fitting α vs. σ curves of P3HT and P3HT/BaTiO3 samples that have different ϵ_r values (Figure S2d), we find that P3HT samples closely fit $\sigma^{-1/8}$, whereas, P3HT/BaTiO3 sample that has a higher ϵ_r and therefore a more Gaussian DOS is best fit with $\sigma^{-1/6}$. We further include the simulated data for ϵ_r = 9 and 12 to show that the slope increases to $\sigma^{-1/5}$ as the DOS becomes more Gaussian in the absence of long-range Coulomb interactions with increasing ϵ_r. Hence, the shape of the α vs. σ curve is determined by the evolving size (Γ_E) and shape (p) of the DOS.

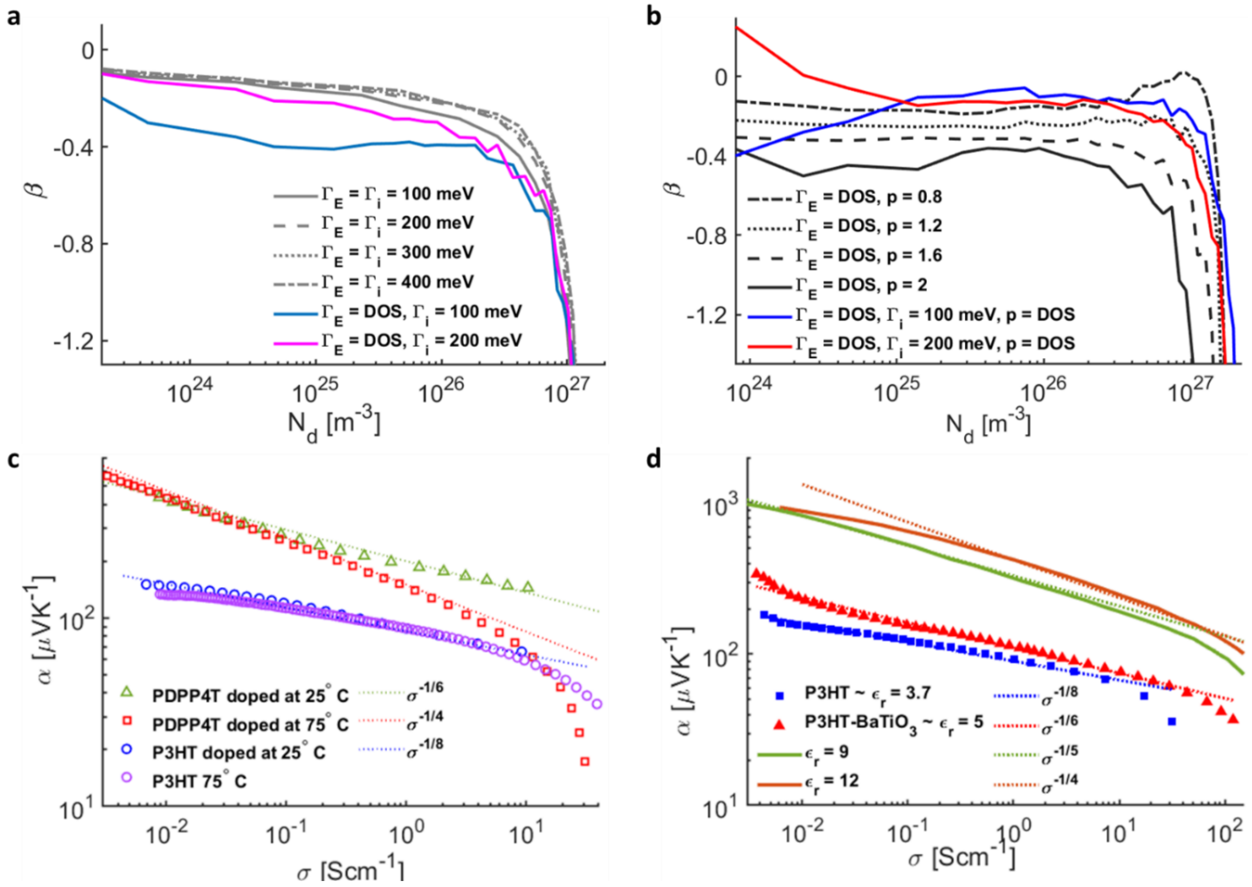

Supplementary Figure S2. The running exponent β of the power-law scaling $\alpha \propto \sigma^{\beta}$ vs. doping concentration. **a** β values extracted from α vs. σ curves for GGD with fixed Γ_E (gray lines) and the α vs. σ curve with Γ_E increasing with doping (pink and cyan lines). p is fixed to 2 for all the cases. **b** β values of α vs. σ curves for a DOS with shape parameter fixed to 0.8, 1.2, 1.6 and 2, and Γ_E increasing with doping (black lines). The blue and red lines show the standard case with DOS computed from Equation (5) in the main text, where Γ_E and shape parameter p are both changing for Γ_i values of 100 and 200 meV respectively. **c** α vs. σ curves of P3HT and PDPP4T obtained from Reference 1 showing that polymers that retain a more Gaussian DOS (homogenous distribution of dopants in PDPP4T doped at 75 °C) exhibit $\alpha \propto \sigma^{-1/4}$ behavior whereas polymers with a more heavy-tailed DOS (heterogenous distribution of dopants) have $\alpha \propto \sigma^{-1/s}$ with s between 6 and 8. **d** P3HT samples closely fit $\sigma^{-1/8}$, whereas P3HT/BaTiO3 sample that has a higher ϵ_r is best fit with $\sigma^{-1/6}$. The simulated data for ϵ_r=9 and 12 is included to show that the slope further increases to $\sigma^{-1/4}$ and $\sigma^{-1/5}$, respectively, as the DOS becomes more Gaussian with the larger ϵ_r.

Experimental Validation and Materials Synthesis

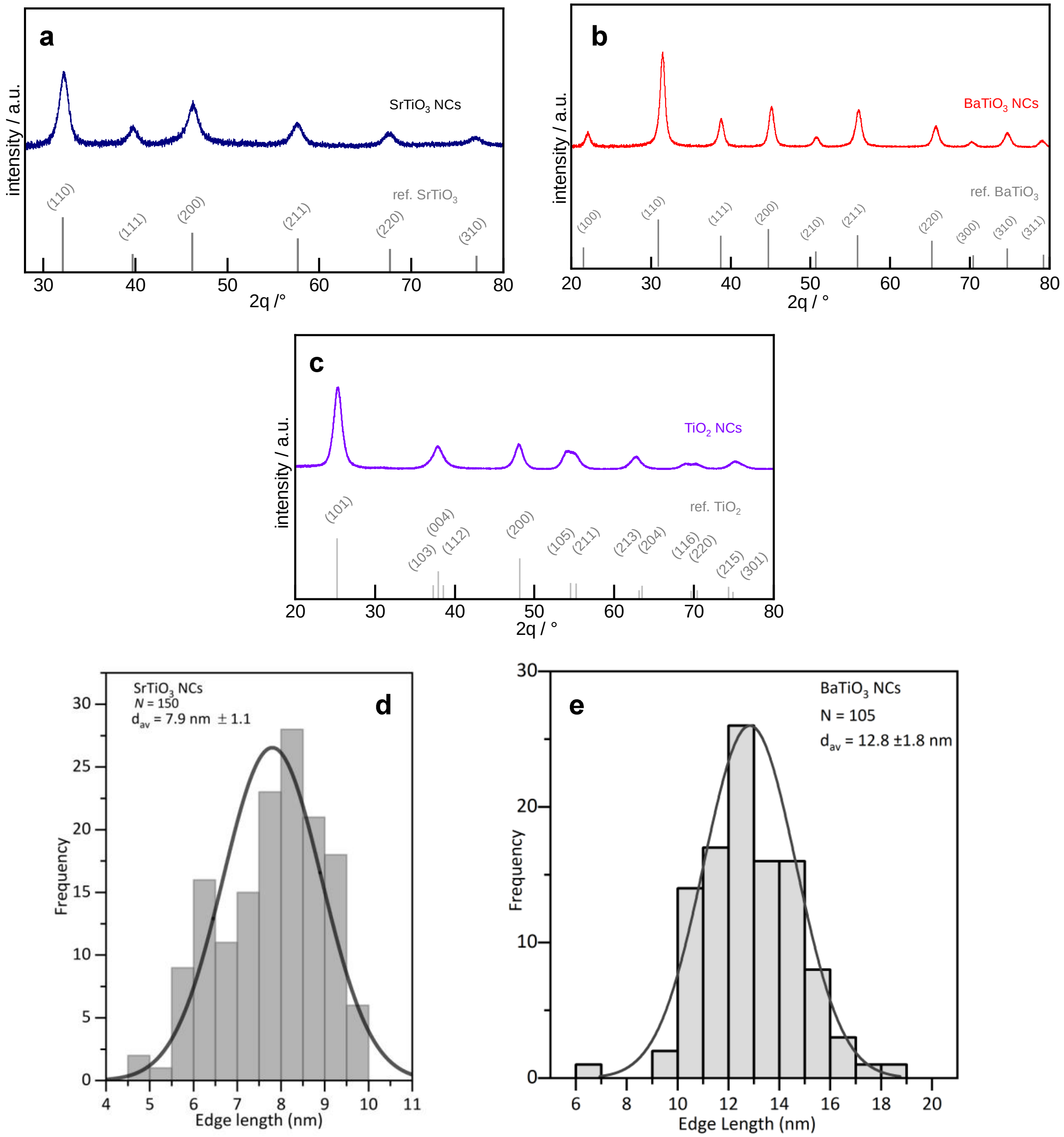

Supplementary Figure S3. Determination of nanocrystal structure and size. X-ray diffraction pattern of **a** $SrTiO_3$, **b** $BaTiO_3$, and **c** TiO_2. Particle size distribution of **d** $SrTiO_3$ and **e** $BaTiO_3$ nanocrystals based on visual analysis of TEM images.

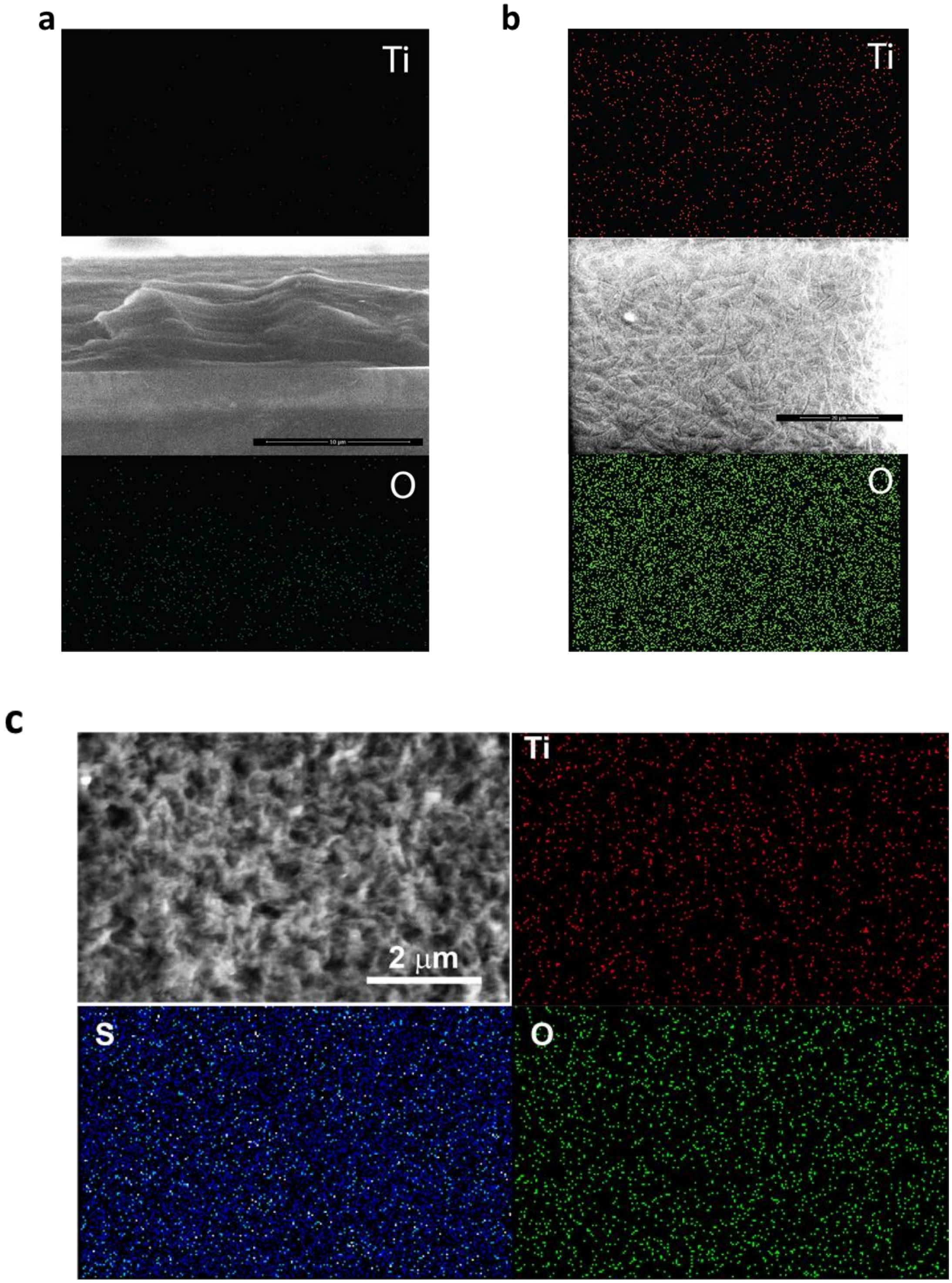

Supplementary Figure S4. Scanning Electron Microscopy – Energy Dispersive Spectroscopy of polymer composites: **a** Cross-sectional scan of TiO_2/P3HT, **b** surface scan of $SrTiO_3$/P3HT and **c** surface scan of BaTiO3/P3HT

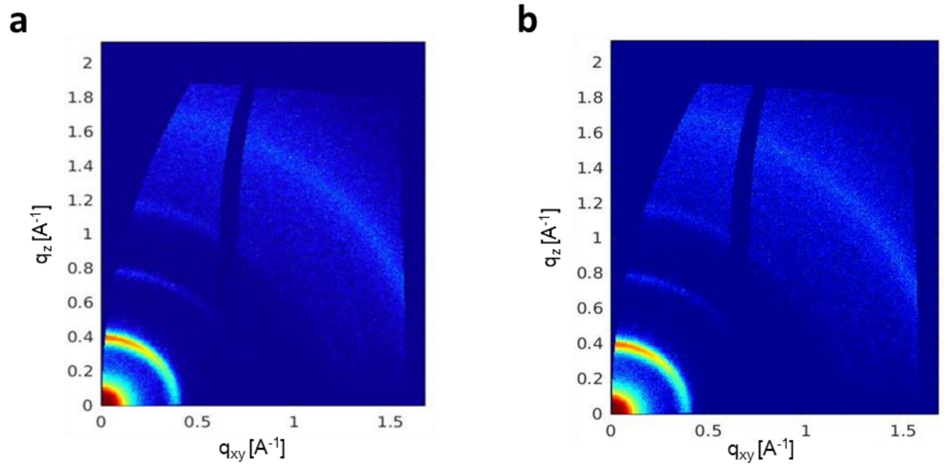

Supplementary Figure S5. GIWAXS pattern of $BaTiO_3$/P3HT composite. **a** The scattering pattern indicates the preservation of signature crystalline peaks with the addition of $BaTiO_3$ nanoparticles when compared to **b** pristine P3HT. The patterns shows the appearance of a prominent (100) peak in the q_z direction.

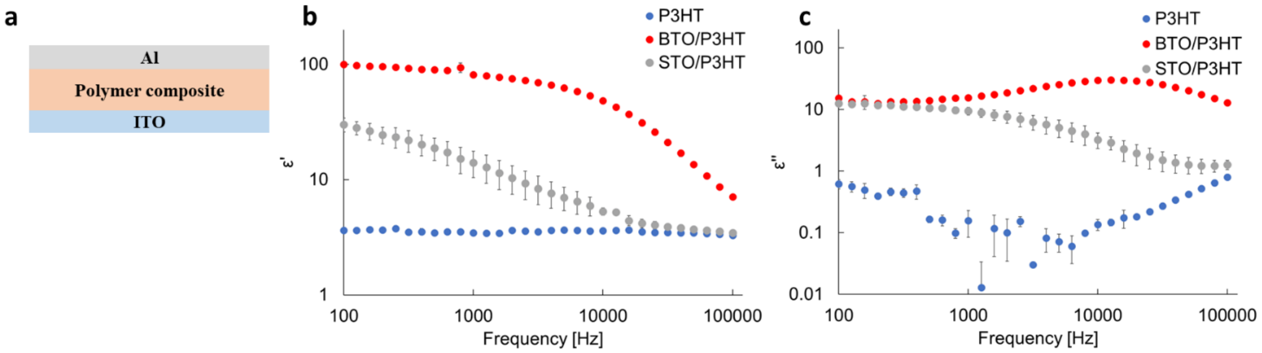

Supplementary Figure S6. Characterization of Dielectric Properties with Impedance Spectroscopy. **a** Device geometry for dielectric spectroscopy study. **b** Dielectric constant vs. frequency plot for different polymer composites. The trend demonstrates a significant effect in the ϵ_{bulk} when additives are added. $BaTiO_3$/P3HT composites shows the highest ϵ followed by STO/P3HT. The error bars represent one standard deviation from duplicate measurements.

Supplementary Note 2:

We investigate the dielectric properties of the composites by preparing devices in a parallel plate capacitor configuration (Supplementary Figure S6a) and measuring their dielectric response with impedance spectroscopy. As seen in Supplementary Figure S6b, the composites exhibit a

significant increase in real permittivity (ϵ') in comparison to the pristine polymer, particularly at low frequencies. A possible explanation for this frequency-dependent phenomenon is termed colossal polarization (CP). CP arises from accumulation of charges at the interfaces. The relevant interfaces in a nanocrystal include internal interfaces, such as grain boundaries and structural defects, as well as external interfaces, such as electrode/nanocrystal and polymer/nanocrystal interfaces. The dispersion of nanocrystals along the polymer matrix can result in a combination of these effects, hence explaining the sudden increase of ϵ'in the composite samples.

The source of high ϵ in ferroelectric oxide nanocrystals such as STO and BTO arises from the slight displacement of the Ti^{4+} ion in the [001] direction inside its octahedral structure in response to an applied electric field.[2] The larger size of the Ba^{2+} ion that causes the cubic structure to expand, extending the Ti-O bond from 1.95 to 2.00 Å and significantly increasing its polarizability and dielectric response.[3]

Power factor enhancement with increasing dielectric constant and the role of energetic disorder

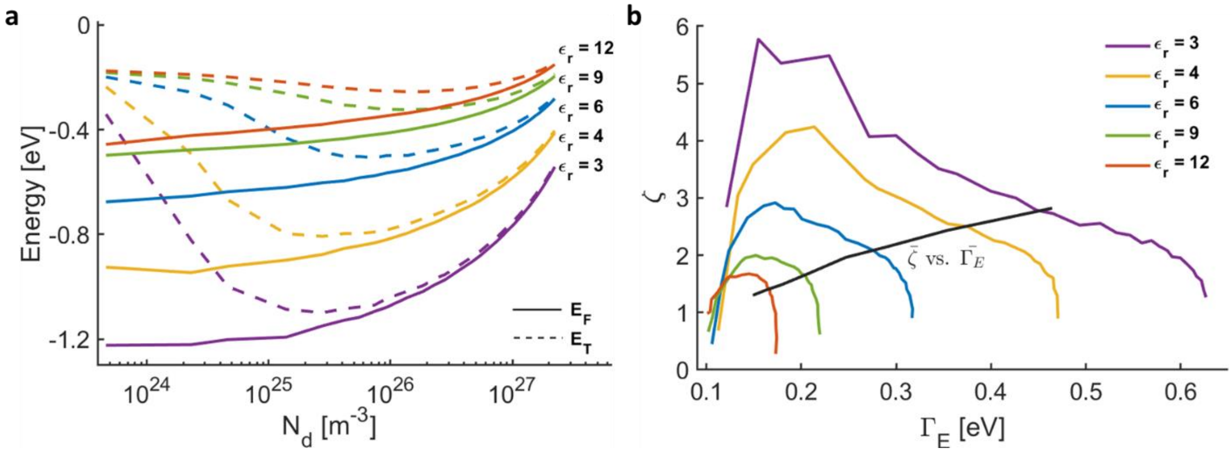

Supplementary Figure S7 a Fermi level E_F and transport energy E_T as a function of doping concentration showing the increase in separation between the transport energy and the Fermi level at higher doping concentration at higher ϵ_r values. **b** The exponent ζ of the power law scaling $\sigma \propto N_d^{\zeta}$ vs. width of the DOS (Γ_E). The average exponent ($\bar{\zeta}$) is proportional to the average energetic disorder ($\overline{\Gamma_E}$).

Supplementary Note 3:

To elucidate the trend of the σ vs. N_d curve and its relation to ϵ_r and energetic disorder Γ_E we plot the running exponent $\zeta = \frac{\mathrm{d}(\ln \sigma)}{\mathrm{d}\,(\ln N_d)}$ vs. Γ_E for ϵ_r ranging from 3 to 12. At lower ϵ_r there is more long-range Coulomb interactions creating deeper traps or heavy tail in the DOS with increasing doping resulting in the higher range of Γ_E in Figure S7b. Charge carriers get 'stuck' in these trap-like states deep in the tail and do not contribute to transport. At higher doping concentrations as the tail states are filled transport improves and hence a much steeper (higher) slope in the σ vs. N_d curve. Hence the slope increases with decreasing ϵ_r due to an increase in Γ_E. The black line in Figure S7b is the average slope value vs. average Γ_E across doping concentrations for a specific $\epsilon_r$value which shows the trend $\sigma \propto N_d^{\zeta}$ with average exponent $\bar{\zeta} \propto \Gamma_E$.

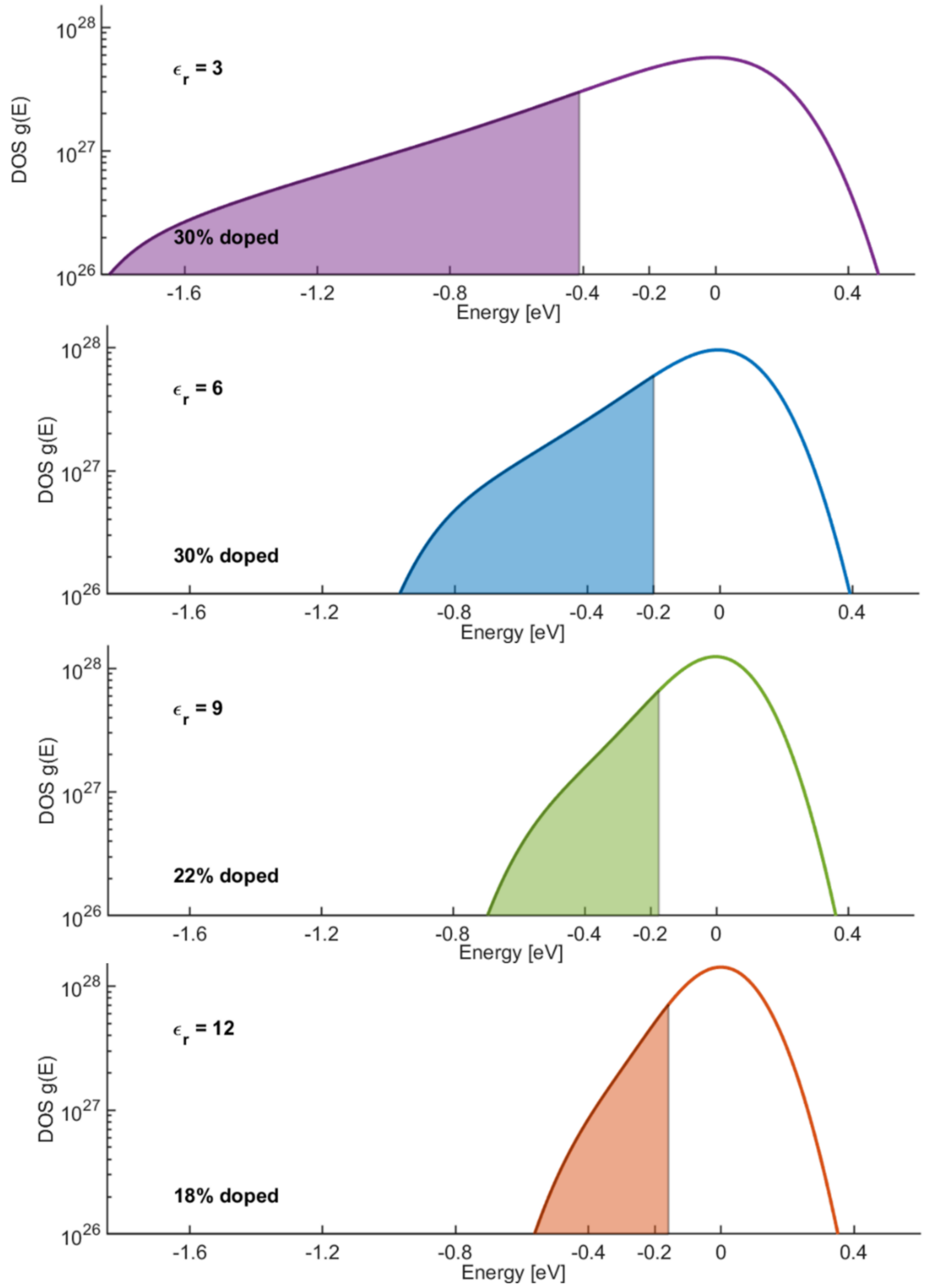

Supplementary Figure S8 DOS with states filled up to the Fermi level E_F corresponding to the doping at which the power factor is maximized at ϵ_r of 3, 6, 9, and 12. At higher ϵ_r values the peak power factor is achieved at lower doping values.

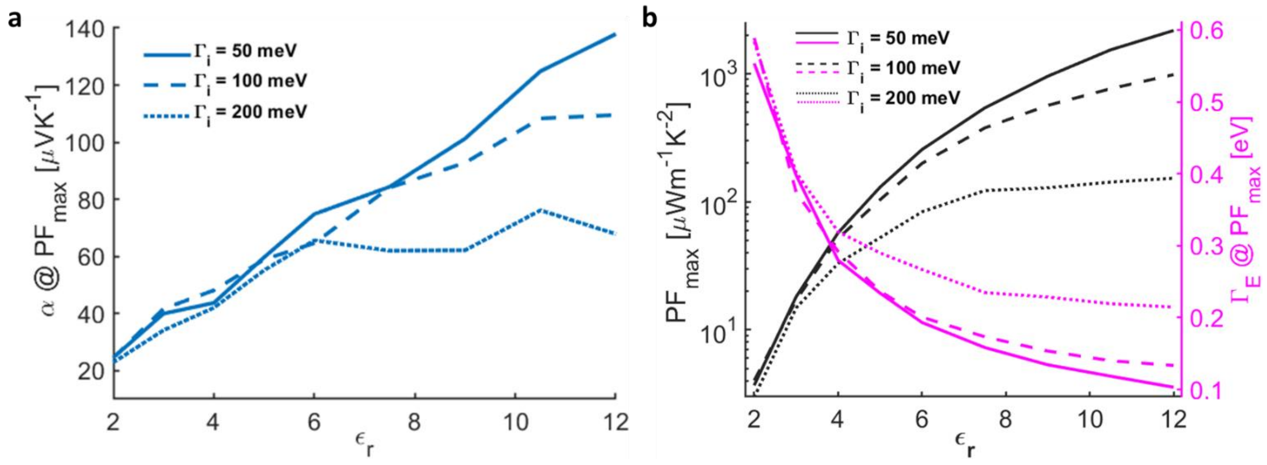

Supplementary Figure S9 a Seebeck coefficient corresponding to the maximum power factor vs. ϵ_r for three different intrinsic disorder Γ_i. We observe an increase in Seebeck due to both the reduction in energetic disorder and the peak power factor achieved at lower doping concentration with increasing ϵ_r. **b** Maximum power factor and the energetic disorder vs. ϵ_r, showing the gain in power factor by changing the material parameters in the simulation (average distance between adjacent sites a = 0.5 nm, overlap factor $\gamma = 0.5$ and dopant radius $r = 3$ Å) to values within the expected range in polymers.

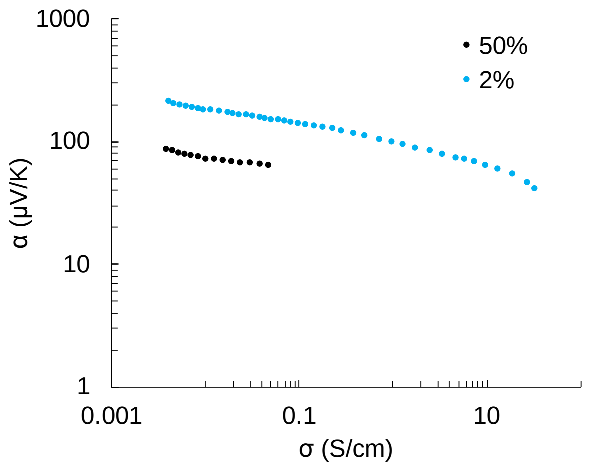

Supplementary Figure S10 Comparison of thermoelectric properties between 2% wt./wt. and 50% wt./wt. $SrTiO_3$ in P3HT. A higher concentration of nanocrystal affects the α vs. σ tradeoff curve.

References:

1. Boyle, C. J. *et al.* Tuning charge transport dynamics via clustering of doping in organic semiconductor thin films. *Nature Communications* **10**, (2019).

2. Kheyrdan, A., Abdizadeh, H., Shakeri, A. & Golobostanfard, M. R. Structural, electrical, and optical properties of sol-gel-derived zirconium-doped barium titanate thin films on transparent conductive substrates. *Journal of Sol-Gel Science and Technology* **86**, 141–150 (2018).

3. Subramanian, M. A., Li, D., Duan, N., Reisner, B. A. & Sleight, A. W. High dielectric constant in ACu3Ti4O12 and ACu3Ti3FeO12 phases. *Journal of Solid State Chemistry* **151**, 323–325 (2000).
